# Protocol for a systematic review of guidelines for rigour in the design, conduct and analysis of biomedical experiments involving laboratory animals

**DOI:** 10.1136/bmjos-2018-000004

**Published:** 2018-09-07

**Authors:** Jan Vollert, Esther Schenker, Malcolm Macleod, Anton Bespalov, Hanno Wuerbel, Martin Christian Michel, Ulrich Dirnagl, Heidrun Potschka, Kimberley E Wever, Thomas Steckler, Bruce Altevogt, Andrew S C Rice, the EQIPD WP3 study group, Judi Clark

**Affiliations:** 1 Pain Research, Department of Surgery and Cancer, Imperial College London, London, UK; 2 Institut de recherches, Servier, Croissy-sur-Seine, France; 3 Centre for Clinical Brain Sciences, University of Edinburgh, Edinburgh, UK; 4 Partnership for Assessment and Accreditation of Scientific Practice, Heidelberg, Germany; 5 Valdman Institute of Pharmacology, Pavlov Medical University, Saint Petersburg, Russia; 6 Division of Animal Welfare, VPH Institute, Vetsuisse Faculty, University of Bern, Bern, Switzerland; 7 Universitätsmedizin Mainz, Johannes-Gutenberg-Universität Mainz, Mainz, Germany; 8 Department of Experimental Neurology, Charité Universitätsmedizin Berlin, Berlin, Germany; 9 Institute of Pharmacology, Toxicology, and Pharmacy, Ludwig-Maximilians- University, Munich, Germany; 10 Department for Health Evidence, Systematic Review Centre for Laboratory Animal Experimentation, Nijmegen Institute for Health Sciences, Radboud university medical centre, Nijmegen, The Netherlands; 11 Janssen Pharmaceutica NV, Beerse, Belgium; 12 Pfizer Inc, New York City, New York, USA

**Keywords:** BMJOS, drug evaluation, preclinical, protocols & guidelines

## Abstract

**Objective:**

Within the last years, there has been growing awareness of the negative repercussions of unstandardized planning, conduct and reporting of preclinical and biomedical research. Several initiatives have set the aim of increasing validity and reliability in reporting of studies and publications, and publishers have formed similar groups. Additionally, several groups of experts across the biomedical spectrum have published experience and opinion-based guidelines and guidance on potential standardized reporting. While all these guidelines cover reporting of experiments, an important step prior to this should be rigours planning and conduction of studies. The aim of this systematic review is to identify and harmonize existing experimental design, conduct and analysis guidelines relating to internal validity and reproducibility of preclinical animal research. The review will also identify literature describing risks of bias pertaining to the design, conduct and analysis of preclinical biomedical research.

**Search strategy:**

PubMed, Embase and Web of Science will be searched systematically to identify guidelines published in English language in peer-reviewed journals before January 2018 (box 1). All articles or systematic reviews in English language that describe or review guidelines on the internal validity and reproducibility of animal studies will be included. Google search for guidelines published on the websites of major funders and professional organisations can be found in (Box 2).

**Screening and annotation:**

Unique references will be screened in two phases: screening for eligibility based on title and abstract, followed by screening for definitive inclusion based on full text. Screening will be performed in SyRF (http://syrf.org.uk). Each reference will be randomly presented to two independent reviewers. Disagreements between reviewers will be resolved by additional screening of the reference by a third, senior researcher.

**Data management and reporting:**

All data, including extracted text and guidelines, will be stored in the SyRF platform. Elements of the included guidelines will be identified using a standardized extraction form. Reporting will follow the PRISMA guidelines as far as applicable.

## Introduction

Within the  last years, there has been growing awareness of the negative repercussions of unstandardised planning, conduct and reporting of preclinical and biomedical research.^1 2^ Several initiatives have set the aim of increasing validity and reliability in reporting of (not only preclinical) studies and publications, such as CAMARADES (Collaborative Approach to Meta-Analysis and Review of Animal Data from Experimental Studies),^3^ NC3Rs (The National Centre for the 3Rs),^4^ SYRCLE (SYstematic Review Center for Laboratory animal Experimentation)^5^ and the EQUATOR (Enhancing the QUAlity and Transparency Of health Research) network.^6^ Publishers have formed similar groups (eg, *The Lancet*’s REWARD (REduce research Waste And Reward Diligence) initiative^7^). Additionally, several experts or groups of experts across the biomedical spectrum, both clinical and preclinical, have published experience and opinion based guidelines and guidance on potential standardised reporting.^8–10^ While many of the points raised are identical or similar between these various guidelines (in fact many experts on the field are part of more than one initiative), they differ in details, rigour and show especially distinct variance in generalisability or specific challenges for a single field. While all these guidelines cover reporting of experiments, an important step prior to this should be rigours planning and conduction of studies, which faces a similar situation.^11^ Consequently, it is hard for researchers to decide which guidelines to follow, especially at the stage of planning future studies.

The aim of this systematic review is to identify and harmonise existing experimental design, conduct and analysis guidelines relating to preclinical animal research. The review will also identify literature describing (either through primary research or systematic review) risks of bias pertaining to the design, conduct and analysis of preclinical biomedical research. Reporting standards will be considered if they refer to topics that are important to consider at planning or experimental, not only at reporting, stage. This review will focus on internal validity and reproducibility of experimental design, conduct and analysis. While we realise that factors like animal housing and welfare are highly important for reproducibility of experiments, they will not be considered in this initial systematic review (SR), which focuses on internal validity. It is planned to analyse the influence of animal care and use at a later point in a separate SR.

Box 1 **Search string**

*Web of Science*

(guideline OR recommendation OR recommendations) AND (“preclinical model” OR “preclinical models” OR “disease model” OR “disease models” OR “animal model” OR “animal models” OR “experimental model” OR “experimental models” OR “preclinical study” OR “preclinical studies” OR “animal study” OR “animal studies” OR “experimental study” OR “experimental studies”)

*PubMed*

((Consensus[mh] OR Consensus development conferences as topic[mh] OR Guidelines as topic [Mesh] OR Practice guidelines as topic[mh] OR guideline[pt] OR practice guideline[pt] OR consensus development conference[pt] OR position statement*[tiab] OR policy statement*[tiab] OR practice parameter*[tiab] OR best practice*[tiab] OR standards[ti] OR guideline[ti] OR guidelines[ti] OR recommendation[ti] OR recommendations[ti]) AND (“Animal Experimentation”[Mesh] OR “Models, Animal”[Mesh] OR Preclinical model[tiab] OR Pre-clinical model[tiab] OR Preclinical models[tiab] OR Pre-clinical models[tiab] OR disease model[tiab] OR disease models[tiab] OR animal model[tiab] OR animal models[tiab] OR experimental model[tiab] OR experimental models[tiab] OR preclinical study[tiab] OR pre-clinical study[tiab] OR preclinical studies[tiab] OR pre-clinical studies[tiab] OR animal study[tiab] OR animal studies[tiab] OR animal experiment*[tiab] OR experimental study[tiab] OR experimental studies[tiab])) OR ((Consensus[mh] OR Consensus development conferences as topic[mh] OR Guidelines as topic [Mesh] OR Practice guidelines as topic[mh] OR guideline[pt] OR practice guideline[pt] OR consensus development conference[pt] OR position statement*[tiab] OR policy statement*[tiab] OR practice parameter*[tiab] OR best practice*[tiab] OR standards[ti] OR guideline[ti] OR guidelines[ti] OR recommendation[ti] OR recommendations[ti]) AND ((Preclinical[tiab] OR Pre-clinical[tiab] OR Experimental[tiab] OR animal[tiab]) AND (Study[tiab] OR Studies[tiab] OR Model[tiab] OR Models[tiab]) AND animals[Mesh:noexp])) OR (((“Methods”[Mesh] OR “methods”[Subheading]) AND (tool[ti] OR protocol[ti])) AND (“Animal Experimentation”[Mesh] OR “Models, Animal”[Mesh] OR ((Preclinical[tiab] OR Pre-clinical[tiab] OR Experimental[tiab] OR animal[tiab]) AND (Study[tiab] OR Studies[tiab] OR Model[tiab] OR Models[tiab])) AND animals[Mesh:noexp])) OR ((position statement*[tiab] OR policy statement*[tiab] OR practice parameter*[tiab] OR best practice*[tiab] OR standards[ti] OR guideline[ti] OR guidelines[ti] OR recommendation[ti] OR recommendations[ti]) AND ((Preclinical[tiab] OR Pre-clinical[tiab] OR Experimental[tiab] OR animal[tiab]) AND (Study[tiab] OR Studies[tiab] OR Model[tiab] OR Models[tiab])) NOT medline[sb])

*Embase*

(Consensus/ or consensus development/ or practice guideline/ or position statement*.ti, ab, kw. OR policy statement*.ti, ab, kw. OR practice parameter*.ti, ab, kw. or best practice*.ti, ab, kw. OR standards.ti. OR guideline.ti. OR guidelines.ti. OR recommendation.ti. OR recommendations.ti.) AND (exp animal experiment/ or exp animal model/ or Preclinical model.ti, ab, kw. OR Pre-clinical model.ti, ab, kw. OR Preclinical models.ti, ab, kw. OR Pre-clinical models.ti, ab, kw. OR disease model.ti, ab, kw. OR disease models.ti, ab, kw. OR animal model.ti, ab, kw. OR animal models.ti, ab, kw. OR experimental model.ti, ab, kw. OR experimental models.ti, ab, kw. OR preclinical study.ti, ab, kw. OR pre-clinical study.ti, ab, kw. OR preclinical studies.ti, ab, kw. OR pre-clinical studies.ti, ab, kw. OR animal study.ti, ab, kw. OR animal studies.ti, ab, kw. OR animal experiment*.ti, ab, kw. OR experimental study.ti, ab, kw. OR experimental studies.ti, ab, kw.) OR ((Consensus/ or consensus development/ or practice guideline/ or position statement*.ti, ab, kw. OR policy statement*.ti, ab, kw. OR practice parameter*.ti, ab, kw. or best practice*.ti, ab, kw. OR standards.ti. OR guideline.ti. OR guidelines.ti. OR recommendation.ti. OR recommendations.ti.) AND ((Preclinical.ti, ab, kw. OR Pre-clinical.ti, ab, kw. OR Experimental.ti, ab, kw. OR animal.ti, ab, kw.) adj2 (Study.ti, ab, kw. OR Studies.ti, ab, kw. OR Model.ti, ab, kw. OR Models.ti, ab, kw.)) AND animal.mp.) OR ((methodology/ or experimental design/ or study design/) and (tool.ti. or protocol.ti.) and (exp animal experiment/ or exp animal model/ or ((Preclinical.ti, ab, kw. OR Pre-clinical.ti, ab, kw. OR Experimental.ti, ab, kw. OR animal.ti, ab, kw.) adj2 (Study.ti, ab, kw. OR Studies.ti, ab, kw. OR Model.ti, ab, kw. OR Models.ti, ab, kw.))) AND animal.mp.)

Box 2 **List of funders and organisations**

*Professional neuroscientific organisations*

Society for Neuroscience (USA)

Cognitive Neuroscience Society (USA)

American College for Neuropsychopharmacology (USA)

Federation of European Neuroscience Societies (EU)

European Brain and Behaviour Society (EU)

European College of Neuropsychopharmacology (EU)

British Neuroscience Association (UK)

*Major funders*

National Institute of Health & Howard Hughes Medical Institute (USA)

Chinese Academy of Sciences & National Natural Sciences Foundation of China (China)

Japan Society for the Promotion of Science & Japan Neuroscience Society (Japan)

European Research Council & Horizon 2020 & Innovative Medicines Initiative (EU)

Wellcome Trust & Medical Research Council (UK)

Deutsche Forschungsgemeinschaft (Germany)

L’agence Nationale de la Recherche & Pasteur Foundation (France)

Dirección General de Investigación Científica y Técnica & Instituto de

Salud Carlos III (Spain)

Ministry of Instruction, Universities, and Research (Italy)

Ministry of Education and Science, Russian Science Foundation and

Russian Foundation for Fundamental Research (Russia)

Ministry of Science and Higher Education (Poland)

Swiss National Science Foundation (Switzerland)

ZonMw (Netherlands)

## Inclusion and exclusion criteria

This study will include all articles or systematic reviews in English language that describe or review guidelines on the internal validity (‘to what extent do the study results reflect a true cause–effect of the intervention?’) and reproducibility of the design, conduct and analysis of preclinical animal studies. Articles that focus strictly focus on toxicity or veterinary drugs only will not be included. Literature not focussing on guidelines, but describing (either through primary research or systematic review) risks of bias pertaining to the design, conduct and analysis of preclinical biomedical research, will also be considered. Although reporting standards are not the key primary objective of this systematic review these will also be searched, screened and extracted, as they can contain useful information that should be considered not only at reporting, but already at planning or experimental stage.

## Screening and annotation

After combining the search results from all sources, potential duplicates or publication of identical guidelines by the same author group in various journals will be identified prior to screening, based on PubMed ID, digital object identifier (DOI) and title, journal and author list. Unique references will then be screened in two phases: (1) screening for eligibility based on title and abstract, followed by (2) screening for definitive inclusion based on full text. Screening will be performed in SyRF (http://syrf.org.uk). Each reference will be randomly presented to two independent reviewers. Reviewers are not blinded to the authors of the presented record. In the first stage, two authors will screen the title and abstract of the retrieved records for eligibility based on predefined inclusion criteria (see above). The title/abstract screening stage will focus on sensitivity (‘could the paper be of any interest?’).

Articles included after the title-abstract screening will undergo concurrent full-text screening for definitive inclusion. We will attempt to obtain full-text versions of all included articles through open access, interlibrary loan or by contacting authors directly. Articles for which no full-text version can be obtained will be excluded from the review.

In both screening stages, disagreements between reviewers will be resolved by additional screening of the reference by a third, senior researcher, who is blind to the individual judgements of the first two reviewers.

## Data management

All references returned from the searches will be downloaded, with entries organised by DOI (if available, or weblink alternatively), publication date, and title. All data, including extracted text and guidelines, will be stored in the SyRF platform.

## Study quality, meta-analysis and risk of bias assessment

These typical stages of systematic reviews are not relevant for this study, as it focusses on guidelines rather than experimental data.

However, both reviewers will rate extracted guidelines rated based on the following system (Ia being the lowest level of provenance, IIIb being the highest):

- Recommendations of individuals or small groups of individuals based on individual experience only
  - Published stand alone.
  - Endorsed or initiated by at least one publisher or scientific society.
- Recommendations by groups of individuals, including a Delphi process
  - Published stand alone.
  - Endorsed or initiated by at least one publisher or scientific society.
- Recommendations based on a systematic review
  - Published stand alone.
  - Endorsed or initiated by at least one publisher or scientific society.

Across guidelines, the elements will be ranked based on the frequency of appearance across the included guidelines.

## Reporting

Elements of the included guidelines will be identified using the extraction form from box 3. Additionally, reporting will follow the Preferred Reporting Items for Systematic Review and Meta-analysis guidelines as far as applicable.

Box 3 **Extraction form**

1. Matching or balancing treatment allocation of animals.

2. Matching or balancing sex of animals across groups.

3. Standardised handling of animals.

4. Randomised allocation of animals to treatment.

5. Randomisation for analysis.

6. Randomised distribution of animals in the animal facilities.

7. Monitoring emergence of confounding characteristics in animals.

8. Specification of unit of analysis.

9. Addressing confounds associated with anaesthesia or analgesia.

10. Selection of appropriate control groups.

11. Concealed allocation of treatment.

12. Study of dose–response relationships.

13. Use of multiple time points measuring outcomes.

14. Consistency of outcome measurement.

15. Blinding of outcome assessment.

16. Establishment of primary and secondary end points.

17. Precision of effect size.

18. Management of conflicts of interest.

19. Choice of statistical methods for inferential analysis.

20. Recording of the flow of animals through the experiment.

21. A priori statements of hypothesis.

22. Choice of sample size.

23. Addressing confounds associated with treatment.

24. Characterisation of animal properties at baseline.

25. Optimisation of complex treatment parameters.

26. Faithful delivery of intended treatment.

27. Degree of characterisation and validity of outcome.

28. Treatment response along mechanistic pathway.

29. Assessment of multiple manifestations of disease phenotype.

30. Assessment of outcome at late/relevant time points.

31. Addressing treatment interactions with clinically relevant comorbidities.

32. Use of validated assay for molecular pathways assessment.

33. Definition of outcome measurement criteria.

34. Comparability of control group characteristics to those of previous studies.

35. Reporting on breeding scheme.

36. Reporting on genetic background.

37. Replication in different models of the same disease.

38. Replication in different species or strains.

39. Replication at different ages.

40. Replication at different levels of disease severity.

41. Replication using variations in treatment.

42. Independent replication.

43. Addressing confounds associated with experimental setting.

44. Addressing confounds associated with setting.

45. Preregistration of study protocol and analysis procedures.

46. Pharmacokinetics to support treatment decisions.

47. Definition of treatment.

48. Interstudy standardisation of end point choice.

49. Define programmatic purpose of research.

50. Interstudy standardisation of experimental design.

51. Research within multicentre consortia.

52. Critical appraisal of literature or systematic review during design phrase.

53. (Multiple) free text.

## Supplementary Material

Reviewer comments

Author's manuscript
