# Supplementary material for: Protocol for a systematic review of guidelines for rigour in the design, conduct and analysis of biomedical experiments involving laboratory animals
Source: BMJ Open Sci. 2018 Sep 7;2(1):e000004. doi: 10.1136/bmjos-2018-000004 (PMC8749326; doi:10.1136/bmjos-2018-000004)
Supplement: Author's manuscript [file bmjos-2018-000004.draft_revisions.pdf]

BMJ Open Science

BMJ Open Science is committed to open peer review. As part of this commitment we make the peer review history of every article we publish publicly available.

When an article is published we post the peer reviewers' comments and the authors' responses online. We also post the versions of the paper that were used during peer review. These are the versions that the peer review comments apply to.

The versions of the paper that follow are the versions that were submitted during the peer review process. They are not the versions of record or the final published versions. They should not be cited or distributed as the published version of this manuscript.

BMJ Open Science is an open access journal and the full, final, typeset and author-corrected version of record of the manuscript is available on our site with no access controls, subscription charges or pay-per-view fees (http://openscience.bmj.com).

If you have any questions on BMJ Open's open peer review process please email info.bmjos@bmj.com

A systematic review of guidelines for rigour in the design, conduct and analysis of biomedical experiments involving laboratory animals.

Authors: Jan Vollert (1), Esther Schenker (2), Malcolm Macleod (3), Anton Bespalov (4,5), Hanno Wuerbel (6), Martin C Michel (4,7), Ulrich Dirnagl (8), Heidrun Potschka (9), Kimberley E Wever (10), Thomas Steckler (11), Bruce Altevogt (12) and Andrew SC Rice (1) on behalf of the EQIPD WP3 study group

1 Pain Research, Department of Surgery and Cancer, Imperial College London, London, UK

2 Institut de Recherches Servier, Croissy-sur- Seine, France

3 Centre for Clinical Brain Sciences, University of Edinburgh, UK

4 Partnership for Assessment and Accreditation of Scientific Practice, Heidelberg, Germany

5 Valdman Institute of Pharmacology, Pavlov Medical University, St. Petersburg, Russia

6 Division of Animal Welfare, VPH Institute, Vetsuisse Faculty, University of Bern, Bern, Switzerland

7 Universitätsmedizin Mainz, Johannes-Gutenberg-Universität Mainz, Mainz, Germany

8 Department of Experimental Neurology, Charité Universitätsmedizin Berlin, Germany

9 Institute of Pharmacology, Toxicology, and Pharmacy, Ludwig-Maximilians- University, Munich, Germany

10 Systematic Review Centre for Laboratory Animal Experimentation, Department for Health Evidence, Nijmegen Institute for Health Sciences, Radboud university medical centre, Nijmegen, The Netherlands

11 Janssen Pharmaceutica NV, Beerse, Belgium

12 Pfizer Inc.

Background and aims

Within the last years, there has been growing awareness of the negative repercussions of unstandardized planning, conduct and reporting of preclinical research [1, 2]. Several initiatives have set the aim of increasing validity and reliability in reporting of (not only preclinical) studies and publications, such as CAMARADES [3], NC3Rs [4], SYRCLE [5] and the EQUATOR network [6]. Publishers have formed similar groups (e.g. The Lancet's REWARD initiative [7]). Additionally, several experts or groups of experts across the biomedical spectrum, both clinical and preclinical, have published experience and opinion based guidelines and guidance on potential standardized reporting [8–10]. While many of the points raised are identical or similar between these various guidelines (in fact many experts on the field are part of more than one initiative), they differ in details, rigour, and show especially distinct variance in generalizability or specific challenges for a single field. While all these guidelines cover reporting of experiments, an important step prior to this should be rigours planning and conduction of studies, which faces a similar situation [11]. Consequently, it is hard for researchers to decide which guidelines to follow, especially at the stage of planning future studies.

The aim of this systematic review is to identify existing experimental design, conduct and analysis guidelines and associated reporting standards relating to preclinical animal research. The review will also identify literature describing (either through primary research or systematic review) the prevalence and impact of risks of bias pertaining to the design, conduct and analysis and reporting of preclinical biomedical research. This review will focus on internal validity of experimental design, conduct and analysis. While we realize that factors like animal housing and welfare are highly important for reproducibility of experiments, they will not be considered in this initial SR, which focuses on internal validity. It is planned to analyse the influence of animal care and use at a later point in a separate SR.

Search strategy

PubMed, Embase and Web of Science will be searched systematically to identify guidelines published in English language in peer-reviewed journals before January 2018, using the search string found in Appendix A. Additional studies will be identified by searching the references of the included articles. As many of the researchers participating in this project are experts on the field of standardization, they will be contacted personally to submit in relevant publications, which will be included additionally, if not identified in the systematic approach. In addition, to capture standards set by funders or organisations that are not (or not yet) published, we will perform a customized google

search for guidelines published on the websites of major funders and professional organisations, listed in Appendix B.

Inclusion and exclusion criteria

This study will include all articles or systematic reviews in English language that describe or review guidelines on validity or the reliability or both of the design, conduct and analysis of preclinical animal studies. Articles that focus on toxicity or veterinary drugs only will not be included. Although reporting standards are not the key primary objective of this systematic review these will also be searched, screened and extracted as a side project, as they can contain useful information with regards to the research question.

Screening and annotation

After combining the search results from all sources, potential duplicates or publication of identical guidelines by the same author group in various journals will be identified prior to screening, based on PubMed ID, DOI, and title, journal and author list. Unique references will then be screened in two phases: 1) screening for eligibility based on title and abstract, followed by 2) screening for definitive inclusion based on full text. Screening will be performed in SyRF (http://syrf.org.uk). Each reference will be randomly presented to two independent reviewers. Reviewers are not blinded to the authors of the presented record. In the first stage, two authors will screen the title and abstract of the retrieved records for eligibility based on predefined inclusion criteria (see below). The title/abstract screening stage will focus on sensitivity ("could the paper be of any interest?").

Articles included after the title-abstract screening will undergo concurrent full-text screening for definitive inclusion. We will attempt to obtain full-text versions of all included articles through open access, interlibrary loan, or by contacting authors directly. Articles for which no full-text version can be obtained will be excluded from the review.

In both screening stages, disagreements between reviewers will be resolved by additional screening of the reference by a third, senior researcher, who is blind to the individual judgements of the first two reviewers.

Data management

All references returned from the searches will be downloaded, with entries organized by DOI (if available, or weblink alternatively), publication date, and title. All data will be stored in the SyRF platform.

Study quality, meta-analysis, and risk of bias assessment

These typical stages of systematic reviews are not relevant for this study, as it focusses on guidelines rather than experimental data.

However, provenance of suggested guidelines will be rated based on the following system:

I. Recommendations of individuals or small groups of individuals based on individual experience only
    a. Published stand-alone
    b. Endorsed or initiated by at least one publisher or scientific society
II. Recommendations by groups of individuals, including a Delphi process
    a. Published stand-alone
    b. Endorsed or initiated by at least one publisher or scientific society
III. Recommendations based on a systematic review
    a. Published stand-alone
    b. Endorsed or initiated by at least one publisher or scientific society

Reporting

Elements of the included guidelines will be identified using the extraction form from Appendix C. Across guidelines, the elements will be ranked based on the frequency of appearance across the included guidelines. Additionally, reporting will follow the PRISMA guidelines as far as applicable.

Acknowledgment

This work is part of the European Quality In Preclinical Data (EQIPD) consortium. This project has received funding from the Innovative Medicines Initiative 2 Joint Undertaking under grant agreement No 777364. This Joint Undertaking receives support from the European Union's Horizon 2020 research and innovation programme and EFPIA. The EQIPD WP3 study group members are: Jan Vollert, Esther Schenker, Malcolm Macleod, Judi Clark, Emily Sena, Anton Bespalov, Bruno

Boulanger, Gernot Riedel, Bettina Platt, Annesha Sil, Martien J Kas, Hanno Wuerbel, Bernhard Voelkl, Martin C Michel, Mathias Jucker, Bettina M Wegenast-Braun, Ulrich Dirnagl, René Bernard, Esmeralda Heiden, Heidrun Potschka, Ann-Marie Waldron, Maarten Loos, Kimberley E Wever, Merel Ritskes-Hoitinga, Tom Van De Casteele, Thomas Steckler, Pim Drinkenburg, Juan Diego Pita Almenar, David Gallacher, Henk Van Der Linde, Anja Gilis, Greet Teuns, Karsten Wicke, Sabine Grote, Bernd Sommer, Janet Nicholson, Sanna Janhunen, Sami Virtanen, Bruce Altevogt, Kristin Cheng, Sylvie Ramboz, Emer Leahy, Isabel A Lefevre, Fiona Ducrey, Javier Guillen, Patri Vergara, Thomas Ingraham and Andrew SC Rice.

REFERENCES

1 Prinz F, Schlange T, Asadullah K. Believe it or not: How much can we rely on published data on potential drug targets? *Nat Rev Drug Discov* 2011;10(9):712.

2 Kilkenny C, Parsons N, Kadyszewski E, et al. Survey of the quality of experimental design, statistical analysis and reporting of research using animals. *PLoS ONE* 2009;4(11):e7824.

3 Hirst JA, Howick J, Aronson JK, et al. The need for randomization in animal trials: An overview of systematic reviews. *PLoS ONE* 2014;9(6):e98856.

4 Du Sert NP, Bamsey I, Bate ST, et al. The Experimental Design Assistant. *Nat Methods* 2017;14(11):1024–25.

5 Hooijmans CR, Rovers MM, Vries RBM de, et al. SYRCLE's risk of bias tool for animal studies. *BMC Med Res Methodol* 2014;14:43.

6 Simera I, Moher D, Hirst A, et al. Transparent and accurate reporting increases reliability, utility, and impact of your research: Reporting guidelines and the EQUATOR Network. *BMC Med* 2010;8:24.

7 Macleod MR, Michie S, Roberts I, et al. Biomedical research: Increasing value, reducing waste. *Lancet* 2014;383(9912):101–04.

8 Hooijmans CR, Leenaars M, Ritskes-Hoitinga M. A gold standard publication checklist to improve the quality of animal studies, to fully integrate the Three Rs, and to make systematic reviews more feasible. *Altern Lab Anim* 2010;38(2):167–82.

9 Smith AJ, Clutton RE, Lilley E, et al. PREPARE: Guidelines for planning animal research and testing. *Lab Anim* 2017:23677217724823.

10 Kilkenny C, Browne WJ, Cuthill IC, et al. Improving bioscience research reporting: The ARRIVE guidelines for reporting animal research. *PLoS Biol* 2010;8(6):e1000412.

11 Henderson VC, Kimmelman J, Fergusson D, et al. Threats to validity in the design and conduct of preclinical efficacy studies: A systematic review of guidelines for in vivo animal experiments. *PLoS Med* 2013;10(7):e1001489.

Appendix A – Search String

Web of Science
(guideline OR recommendation OR recommendations) AND
("preclinical model" OR "preclinical models" OR "disease model" OR "disease models" OR "animal model" OR "animal models" OR "experimental model" OR "experimental models" OR "preclinical study" OR "preclinical studies" OR "animal study" OR "animal studies" OR "experimental study" OR "experimental studies")

Pubmed
((Consensus[mh] OR Consensus development conferences as topic[mh] OR Guidelines as topic [Mesh] OR Practice guidelines as topic[mh] OR guideline[pt] OR practice guideline[pt] OR consensus development conference[pt] OR position statement*[tiab] OR policy statement*[tiab] OR practice parameter*[tiab] OR best practice*[tiab] OR standards[ti] OR guideline[ti] OR guidelines[ti] OR recommendation[ti] OR recommendations[ti]) AND ("Animal Experimentation"[Mesh] OR "Models, Animal"[Mesh] OR Preclinical model[tiab] OR Pre-clinical model[tiab] OR Preclinical models[tiab] OR Pre-clinical models[tiab] OR disease model[tiab] OR disease models[tiab] OR animal model[tiab] OR animal models[tiab] OR experimental model[tiab] OR experimental models[tiab] OR preclinical study[tiab] OR pre-clinical study[tiab] OR preclinical studies[tiab] OR pre-clinical studies[tiab] OR animal study[tiab] OR animal studies[tiab] OR animal experiment*[tiab] OR experimental study[tiab] OR experimental studies[tiab])) OR ((Consensus[mh] OR Consensus development conferences as topic[mh] OR Guidelines as topic [Mesh] OR Practice guidelines as topic[mh] OR guideline[pt] OR practice guideline[pt] OR consensus development conference[pt] OR position statement*[tiab] OR policy statement*[tiab] OR practice parameter*[tiab] OR best practice*[tiab] OR standards[ti] OR guideline[ti] OR guidelines[ti] OR recommendation[ti] OR recommendations[ti]) AND ((Preclinical[tiab] OR Pre-clinical[tiab] OR Experimental[tiab] OR animal[tiab]) AND (Study[tiab] OR Studies[tiab] OR Model[tiab] OR Models[tiab]) AND animals[Mesh:noexp])) OR ((("Methods"[Mesh] OR "methods"[Subheading]) AND (tool[ti] OR protocol[ti])) AND ("Animal Experimentation"[Mesh] OR "Models, Animal"[Mesh] OR ((Preclinical[tiab] OR Pre-clinical[tiab] OR Experimental[tiab] OR animal[tiab]) AND (Study[tiab] OR Studies[tiab] OR Model[tiab] OR Models[tiab])) AND animals[Mesh:noexp])) OR ((position statement*[tiab] OR policy statement*[tiab] OR practice parameter*[tiab] OR best practice*[tiab] OR standards[ti] OR guideline[ti] OR guidelines[ti] OR recommendation[ti] OR recommendations[ti]) AND ((Preclinical[tiab] OR Pre-clinical[tiab] OR Experimental[tiab] OR animal[tiab]) AND (Study[tiab] OR Studies[tiab] OR Model[tiab] OR Models[tiab])) NOT medline[sb])

EMBASE

(Consensus/ or consensus development/ or practice guideline/ or position statement*.ti,ab,kw. OR policy statement*.ti,ab,kw. OR practice parameter*.ti,ab,kw. or best practice*.ti,ab,kw. OR standards.ti. OR guideline.ti. OR guidelines.ti. OR recommendation.ti. OR recommendations.ti.) AND (exp animal experiment/ or exp animal model/ or Preclinical model.ti,ab,kw. OR Pre-clinical model.ti,ab,kw. OR Preclinical models.ti,ab,kw. OR Pre-clinical models.ti,ab,kw. OR disease model.ti,ab,kw. OR disease models.ti,ab,kw. OR animal model.ti,ab,kw. OR animal models.ti,ab,kw. OR experimental model.ti,ab,kw. OR experimental models.ti,ab,kw. OR preclinical study.ti,ab,kw. OR pre-clinical study.ti,ab,kw. OR preclinical studies.ti,ab,kw. OR pre-clinical studies.ti,ab,kw. OR animal study.ti,ab,kw. OR animal studies.ti,ab,kw. OR animal experiment*.ti,ab,kw. OR experimental study.ti,ab,kw. OR experimental studies.ti,ab,kw.) OR ((Consensus/ or consensus development/ or practice guideline/ or position statement*.ti,ab,kw. OR policy statement*.ti,ab,kw. OR practice parameter*.ti,ab,kw. or best practice*.ti,ab,kw. OR standards.ti. OR guideline.ti. OR guidelines.ti. OR recommendation.ti. OR recommendations.ti.) AND ((Preclinical.ti,ab,kw. OR Pre-clinical.ti,ab,kw. OR Experimental.ti,ab,kw. OR animal.ti,ab,kw.) adj2 (Study.ti,ab,kw. OR Studies.ti,ab,kw. OR Model.ti,ab,kw. OR Models.ti,ab,kw.)) AND animal.mp.) OR ((methodology/ or experimental design/ or study design/) and (tool.ti. or protocol.ti.) and (exp animal experiment/ or exp animal model/ or ((Preclinical.ti,ab,kw. OR Pre-clinical.ti,ab,kw. OR Experimental.ti,ab,kw. OR animal.ti,ab,kw.) adj2 (Study.ti,ab,kw. OR Studies.ti,ab,kw. OR Model.ti,ab,kw. OR Models.ti,ab,kw.))) AND animal.mp.)

Appendix B – List of funders and organisations

Professional neuroscientific organizations:

Society for Neuroscience (US)

Cognitive Neuroscience Society (US)

American College for Neuropsychopharmacology (US)

Federation of European Neuroscience Societies (EU)

European Brain and Behaviour Society (EU)

European College of Neuropsychopharmacology (EU)

British Neuroscience Association (UK)

Major funders:

US - National Institute of Health & Howard Hughes Medical Institute

China - Chinese Academy of Sciences & National Natural Sciences Foundation of China

Japan - Japan Society for the Promotion of Science & Japan Neuroscience Society

EU - European Research Council & Horizon 2020 & Innovative Medicines Initiative

UK - Wellcome Trust & Medical Research Council

Germany - Deutsche Forschungsgemeinschaft

France - L'agence Nationale de la Recherche & Pasteur Foundation

Spain - Dirección General de Investigación Científica y Técnica & Instituto de Salud Carlos III

Italy - Ministry of Instruction, Universities, and Research

Russia - Ministry of Education and Science & Russian Science Foundation & Russian Foundation for Fundamental Research

Poland - Ministry of Science and Higher Education

Switzerland - Swiss National Science Foundation

Netherlands - ZonMw

Appendix C – Extraction form

1. Matching or balancing treatment allocation of animals
2. Matching or balancing sex of animals across groups
3. Standardized handling of animals
4. Randomized allocation of animals to treatment
5. Randomization for analysis
6. Randomized distribution of animals in the animal facilities
7. Monitoring emergence of confounding characteristics in animals
8. Specification of unit of analysis
9. Addressing confounds associated with anaesthesia or analgesia
10. Selection of appropriate control groups
11. Concealed allocation of treatment
12. Study of dose-response relationships
13. Use of multiple time points measuring outcomes
14. Consistency of outcome measurement
15. Blinding of outcome assessment
16. Establishment of primary and secondary end points
17. Precision of effect size
18. Management of conflicts of interest
19. Choice of statistical methods for inferential analysis
20. Recording of the flow of animals through the experiment
21. A priori statements of hypothesis
22. Choice of sample size
23. Addressing confounds associated with treatment
24. Characterization of animal properties at baseline
25. Optimization of complex treatment parameters
26. Faithful delivery of intended treatment
27. Degree of characterization and validity of outcome
28. Treatment response along mechanistic pathway
29. Assessment of multiple manifestations of disease phenotype
30. Assessment of outcome at late/relevant time points
31. Addressing treatment interactions with clinically relevant co-morbidities
32. Use of validated assay for molecular pathways assessment
33. Definition of outcome measurement criteria
34. Comparability of control group characteristics to those of previous studies

35. Reporting on breeding scheme
36. Reporting on genetic background
37. Replication in different models of the same disease
38. Replication in different species or strains
39. Replication at different ages
40. Replication at different levels of disease severity
41. Replication using variations in treatment
42. Independent replication
43. Addressing confounds associated with experimental setting
44. Addressing confounds associated with setting
45. Pre-registration of study protocol and analysis procedures
46. Pharmacokinetics to support treatment decisions
47. Definition of treatment
48. Inter-study standardization of end point choice
49. Define programmatic purpose of research
50. Inter-study standardization of experimental design
51. Research within multicentre consortia
52. Critical appraisal of literature or systematic review during design phrase
53. (multiple) free text
