# Supplementary material for: Protocol for a systematic review of guidelines for rigour in the design, conduct and analysis of biomedical experiments involving laboratory animals
Source: BMJ Open Sci. 2018 Sep 7;2(1):e000004. doi: 10.1136/bmjos-2018-000004 (PMC8749326; doi:10.1136/bmjos-2018-000004)
Supplement: Reviewer comments [file bmjos-2018-000004.reviewer_comments.pdf]

PEER REVIEW HISTORY

BMJ Open Science publishes all reviews undertaken for accepted manuscripts. Reviewers are asked to complete a checklist review form (http://openscience.bmj.com/pages/wp-content/uploads/sites/62/2018/04/BMJ-Open-Science-Reviewer-Score-Sheet.pdf) and are provided with free text boxes to elaborate on their assessment. These free text comments are reproduced below.

ARTICLE DETAILS

**TITLE (PROVISIONAL)**	A systematic review of guidelines for rigour in the design, conduct and analysis of biomedical experiments involving laboratory animals.
**AUTHORS**	Jan Vollert (Corresponding Author)<br>Esther Schenker<br>Malcolm Macleod<br>Anton Bespalov<br>Hanno Wuerbel<br>Martin Christian Michel<br>Ulrich Dirnagl<br>Heidrun Potschka<br>Kimberley Wever<br>Thomas Steckler<br>Bruce Altevogt<br>Andrew SC Rice

VERSION 1 - REVIEW

**REVIEWER 1**	Kristina Thayer<br>United States Environmental Protection Agency<br><br>Conflict of Interest: None declared
**REVIEW RETURNED**	20-03-18

**GENERAL COMMENTS**	.Background and aims comment: maybe make more broad - preclinical and biomedical research?<br><br>Search Strategy comment: would it be too broad for you to consider including OECD harmonized guidelines for conducting animal studies in toxicology? that would be a type of gray literature<br><br>Inclusion and exclusion criteria comment: ah, so my previous comment on OECD might be out of scope...but it looks like it might fall into the side project<br><br>Study quality, meta-analysis and risk of bias assessment comment 2nd sentence: this could be a bit clearer perhaps? I'm not sure which you would consider "best available"<br><br>Reporting comment: I'm not sure I see appendix C?

REVIEWER 2	Marc Avey<br>ICF Canada<br><br>Conflict of Interest: None declared
**REVIEW RETURNED**	01-03-18

GENERAL COMMENTS	I have a number of suggestion that I hope will be helpful and more useful at the protocol stage than after the research is conducted. Note: I cannot access the supplemental materials and my emails to BMJ OS have thus far bounced so apologies if this is covered there.<br><br>1. Is the research question or study objective clearly defined?<br><br>“The aim of this systematic review is to identify existing experimental design, conduct and analysis guidelines and associated reporting standards relating to preclinical animal research. The review will also identify literature describing (either through primary research or systematic review) the prevalence and impact of risks of bias pertaining to the design, conduct and analysis and reporting of preclinical biomedical research. This review will focus on internal validity of experimental design, conduct and analysis.”<br><br>There is a lot being covered in the aims and it’s not clear how the search/inclusion/analysis will answer them based on the sections in the protocol. I suggest using the same language in the background with the ‘aims’ and the subsequent sections to make it clearer how they align. In particular, I was unclear how ‘prevalence’ and ‘impact’ were being assessed (inclusion/exclusion talks about validity/reliability; and the analysis talks about provenance and frequency). I’m also unclear as to how the authors intend to focus on internal validity?<br><br>There also appears to be the intention to use this review to harmonize competing guidance but this is not explicitly listed as an aim of the review. If this is part of the aim I think it should be articulated more fully as it impacts how data may collected and analyzed.<br><br>3. Is the study design appropriate to answer the research question …?<br><br>As above, I found the subsequent sections after the initial aims to not clearly link to the aims. Reporting standards are part of the aims but also just a side project? Terminology seems to change from one section to the next (aim vs key objective; prevalence/impact vs validity/reliability vs provenance/frequency). Apologies if this is described in appendix C but I can’t access it.<br><br>Are the outcomes clearly defined?

	Again, the authors switch language in different sections (e.g. aims vs key primary objective?) in the protocol which makes it confusing. It would also be helpful to define what the authors means by prevalence, impact, internal validity and how the analysis relates. I found it unclear how the review is focused on internal validity or the relevance of suggesting that animal housing/welfare is not part of this.<br><br>13. Is the supplementary reporting complete (e.g. ARRIVE checklist, PRISMA checklist, study registration; funding details)?<br><br>I strongly urge the authors (and editors of BMJ Open Science) to use PRISMA-P review: http://www.prisma-statement.org/Extensions/Protocols.aspx This is an evidence-based reporting standard for systematic review protocols. Although the review is labelled a 'systematic review' it appears methodologically to be more appropriately a scoping review by design.<br><br>General Comments:<br><br>Background:<br><br>1. The focus of this review is on internal validity. I assume the authors mean systematic variation?<br><br>Search:<br><br>1. Consider explicitly searching for government design/reporting/analyses standards for these types of experiments.<br><br>2. Consider stating that there will be no start date limit on the search. I cannot access the search itself to review it.<br><br>3. I strongly recommend using the PRESS method to evaluate your search strategy prior to implementing it. It is better to peer review your search prior to implementation and use an evidence-based standardized process conducted by experts (information specialist).<br><br>Inclusion and Exclusion Criteria:<br><br>1. To be clear, you are including guidelines themselves as well as articles/systematic reviews that describe/review guidelines? The purpose of the articles/systematic reviews is to identify guidelines (and maybe reporting standards?).<br><br>2. Should this also not clearly state that you will include literature (primary research/systematic reviews) that asses the prevalence/impact of risk of bias for design/conduct/analysis/reporting. This is the second part of the aim and is different from guidelines/reporting standards. Perhaps I'm being overly pedantic here though.

	3. By ‘both’ you mean all three of design/conduct/analysis?<br><br>4. I think it would help to define validity and reliability here (just internal validity since that is the focus?). I also assume this is for guidelines related to primary studies of in vivo preclinical research?<br><br>5. How will you handle guidelines/reporting standards that apply generally and include toxicity/veterinary uses?<br><br>6. The sentence: “Although reporting standards are not…” is entirely confusing for me. Reporting standards are listed as part of the aim of the review, but here they are a related side project? The language is also confusing because above there was just an aim, and now there is a key primary objective. I would suggest to standardize the language for aims in the above section and only include information about what is relevant to this research proposal. I don’t have access to appendix A, but if reporting standards are not part of this protocol than the search terms relevant for identifying them should be removed.<br><br><br>Screening and Annotation<br><br>“see below” should be “see above”?<br><br>Data Management<br><br>1. Is the data stored in SyRF just the references/PDF or also the extracted data/text?<br><br>Study quality, meta-analysis, and risk of bias assessment<br><br>1. Will this be done in duplicate?<br><br>2. The provenance (not an aim/outcome from above) appears to be a validity assessment. These examples of assessing study quality may be helpful (or not).<br><br>https://www.ncbi.nlm.nih.gov/pubmed/24965222<br><br>http://onlinelibrary.wiley.com/doi/10.1002/14651858.MR000030.pub2/abstract;jsessionid=9C73024C83EB23B20F7EFEECB2BC24CB.f01t01<br><br>3. I see how the rating system above applies to guidelines and/or reporting standards, but what is the plan for the second aim to investigate the prevalence and impact of risk of bias/internal validity? I also don’t see how the author intend to focus (sort?) elements of guidelines into internal validity vs non-internal validity? Apologies if this is described in appendix C but I can’t access it.<br><br>Reporting<br><br>1. The ranking based on frequency of elements in the guidelines is technically part of the analysis and should be in the above section.

	2. “Aadditionally, reporting will follow the PRISMA guidelines as far as applicable.” Yes.

VERSION 1 – AUTHOR RESPONSE

Dear Editor, dear Editor-in-Chief,
Thank you very much for your reply. We are grateful to you, the Section Editor and both reviewers for these very positive evaluations. We have revised the manuscript according to the reviewer's comments. All changes are described en detail per comment below. The modifications of our manuscript are shaded in yellow to allow easy recognition.

Reviewer 1: Background and aims comment: maybe make more broad - preclinical and biomedical research?
Author's response: We agree and have amended the wording.

Search Strategy comment: would it be too broad for you to consider including OECD harmonized guidelines for conducting animal studies in toxicology? that would be a type of gray literature
Inclusion and exclusion criteria comment: ah, so my previous comment on OECD might be out of scope...but it looks like it might fall into the side project
Author's response: Yes, we agree that the OECD harmonized guidelines are beyond the scope of this initial review, but will be of interest for the side project.

Study quality, meta-analysis and risk of bias assessment comment 2nd sentence: this could be a bit clearer perhaps? I'm not sure which you would consider "best available"
Author's response: We thank the reviewer for noting this ambiguity, we have inserted a clarifying statement in the text.

Reporting comment: I'm not sure I see appendix C?
Author's response: The appendix was in the initial submission, but seems to have gone lost on the way, as Reviewer #2 did not find it as well. We made sure it is included this time.

Reviewer 2: I have a number of suggestion that I hope will be helpful and more useful at the protocol stage than after the research is conducted. Note: I cannot access the supplemental materials and my emails to BMJ OS have thus far bounced so apologies if this is covered there.
1. Is the research question or study objective clearly defined?
"The aim of this systematic review is to identify existing experimental design, conduct and analysis guidelines and associated reporting standards relating to preclinical animal research. The review will also identify literature describing (either through primary research or systematic review) the prevalence and impact of risks of bias pertaining to the design, conduct and analysis and reporting of preclinical biomedical research. This review will focus on internal validity of experimental design, conduct and analysis."
There is a lot being covered in the aims and it's not clear how the search/inclusion/analysis will answer them based on the sections in the protocol. I suggest using the same language in the background with the 'aims' and the subsequent sections to make it clearer how they align. In particular, I was unclear how 'prevalence' and 'impact' were being assessed (inclusion/exclusion talks about validity/reliability; and the analysis talks about provenance and frequency). I'm also unclear as to how the authors intend to focus on internal validity?
Author's response: This topic relates to many other questions, which are handled below. We throughout the manuscript now use the terms "internal validity and reproducibility" only. We throughout the manuscript deleted the misleading wording of investigating prevalence and impact of risk of bias. Reporting standards are not part of the aim, which was not phrased carefully enough.

There also appears to be the intention to use this review to harmonize competing guidance but this is not explicitly listed as an aim of the review. If this is part of the aim I think it should be articulated more fully as it impacts how data may collected and analyzed.
Author's response: We agree and have amended the phrasing to "..Aim of this systematic review is to identify and harmonize existing experimental design, conduct and analysis guidelines…"

3. Is the study design appropriate to answer the research question …?
As above, I found the subsequent sections after the initial aims to not clearly link to the aims. Reporting standards are part of the aims but also just a side project? Terminology seems to change

from one section to the next (aim vs key objective; prevalence/impact vs validity/reliability vs provenance/frequency). Apologies if this is described in appendix C but I can't access it.
Author's response: We thank the reviewer to raising attention to this ambiguity. Reporting standards are not part of the aim, which was not phrased carefully enough. The purpose is to identify guidelines on conduction and analysis. We assume that some reporting standards will include information that should be considered rather at the experimental or even planning stage already, and not just at the reporting stage, which is why we will look at reporting standards. We phrased this more clearly throughout the protocol (see above and below as well).

Are the outcomes clearly defined?
Again, the authors switch language in different sections (e.g. aims vs key primary objective?) in the protocol which makes it confusing. It would also be helpful to define what the authors' mean by prevalence, impact, internal validity and how the analysis relates. I found it unclear how the review is focused on internal validity or the relevance of suggesting that animal housing/welfare is not part of this.
Author's response: We throughout the manuscript now use the terms "internal validity and reproducibility" only. We found that animal housing and welfare are best placed under a different domain than the experimental conduct and analysis, as it is a big body of literature on its own (see below as well).

13. Is the supplementary reporting complete (e.g. ARRIVE checklist, PRISMA checklist, study registration; funding details)?
I strongly urge the authors (and editors of BMJ Open Science) to use PRISMA-P review: http://www.prisma-statement.org/Extensions/Protocols.aspx This is an evidence-based reporting standard for systematic review protocols. Although the review is labelled a 'systematic review' it appears methodologically to be more appropriately a scoping review by design.
Author's response: While we generally agree that PRISMA-P is an important tool for systematic review protocols, it has been developed for what systematic reviews are mostly conducted in, which is clinical studies. This being a systematic review (systematic in the meaning of being based on a systematic, reproducible database search) of guidelines rather than outcomes we found some items to be not applicable for this particular protocol. Generally, we followed PRISMA-P as much as possible.

General Comments:
Background:
1. The focus of this review is on internal validity. I assume the authors mean systematic variation?
Author's response: Aim of the review is to find elements that relate to the question "to what extent do the study results reflect a true cause-effect of the intervention?" (what we consider internal validity, which is threatened by bias, i.e. systematic error), rather than to the question "can the study results be generalized to other studies / the population / patients /…?" (what we would consider external validity, threatened by indirectness). We tried to phrase more clearly throughout the manuscript (see above and below as well).

Search:
1. Consider explicitly searching for government design/reporting/analyses standards for these types of experiments.
Author's response: We agree that this is an important issue, which is why we explicitly search on the websites of major societies and funders as listed in Appendix B, which covers major governmental funding organizations.

2. Consider stating that there will be no start date limit on the search. I cannot access the search itself to review it.
Author's response: We agree and have amended.

3. I strongly recommend using the PRESS method to evaluate your search strategy prior to implementing it. It is better to peer review your search prior to implementation and use an evidence-based standardized process conducted by experts (information specialist).

Author's response: We agree that optimizing the search string is an important topic, and have done so with an information specialist. Hopefully, the reviewer will be able to find our search strings in the appendix in the revised submission.

Inclusion and Exclusion Criteria:
1. To be clear, you are including guidelines themselves as well as articles/systematic reviews that describe/review guidelines? The purpose of the articles/systematic reviews is to identify guidelines (and maybe reporting standards?).
Author's response: The purpose is to identify guidelines on conduction and analysis, and we will therefore also include systematic reviews that report on guidelines (and potentially give recommendations).

2. Should this also not clearly state that you will include literature (primary research/systematic reviews) that asses the prevalence/impact of risk of bias for design/conduct/analysis/reporting. This is the second part of the aim and is different from guidelines/reporting standards. Perhaps I'm being overly pedantic here though.
Author's response: We agree that these parts were not harmonized well enough. A sentenced is added here on including primary research as well.

3. By 'both' you mean all three of design/conduct/analysis?
Author's response: "both" aimed at "both validity and reliability", we phrased more clearly and changed to the terms now used throughout the manuscript, "internal validity and reproducibility" (see above and below as well).

4. I think it would help to define validity and reliability here (just internal validity since that is the focus?). I also assume this is for guidelines related to primary studies of in vivo preclinical research?
Author's response: This is indeed not an easy topic, and we have intensive discussions within our group on these questions. As a matter of fact, many items are not clearly and easily sorted to either internal or reproducibility or external validity, but may be considered gray areas. We throughout the manuscript deleted the misleading wording of investigating prevalence and impact of risk of bias. We throughout the manuscript now use the terms "internal validity and reproducibility". Aim of the review is to find elements that are linked to the question "to what extent do the study results reflect a true cause-effect of the intervention?", rather than to the question "can the study results be generalized to other studies / the population / patients /…?". We tried to phrase more clearly (see above and below as well).

5. How will you handle guidelines/reporting standards that apply generally and include toxicity/veterinary uses?
Author's response: In these cases, the guidelines would be considered, only specifically toxicity/veterinary only cases are excluded. We phrased more carefully.

6. The sentence: "Although reporting standards are not…" is entirely confusing for me. Reporting standards are listed as part of the aim of the review, but here they are a related side project? The language is also confusing because above there was just an aim, and now there is a key primary objective. I would suggest to standardize the language for aims in the above section and only include information about what is relevant to this research proposal. I don't have access to appendix A, but if reporting standards are not part of this protocol than the search terms relevant for identifying them should be removed.
Author's response: The purpose is to identify guidelines on conduct and analysis. We assume that some reporting standards will include information that should be considered rather at the experimental or even planning stage already, and not just at the reporting stage, which is why we will look at reporting standards. We phrased this more clearly throughout the protocol (see above as well).

Screening and Annotation
"see below" should be "see above"?
Author's response: Thank you for noting, yes, was corrected to "see above".

Data Management

1. Is the data stored in SyRF just the references/PDF or also the extracted data/text?
Author's response: We thank the reviewer for noting this ambiguity, all data, including full extracted guidelines and text will be stored in SyRF. We amended the text accordingly.

Study quality, meta-analysis, and risk of bias assessment
1. Will this be done in duplicate?
Author's response: Yes it will, we have clarified so in the text.

2. The provenance (not an aim/outcome from above) appears to be a validity assessment. These examples of assessing study quality may be helpful (or not).
https://www.ncbi.nlm.nih.gov/pubmed/24965222
http://onlinelibrary.wiley.com/doi/10.1002/14651858 Add to Citavi project by DOI.MR000030.pub2/abstract;jsessionid=9C73024C83EB23B20F7EFEECB2BC24CB.f01t01
Author's response: We thank the reviewer for this literature, which will be of interest for the study. The provenance at this stage will not be a validity assessment, but rather a method of documentation.

3. I see how the rating system above applies to guidelines and/or reporting standards, but what is the plan for the second aim to investigate the prevalence and impact of risk of bias/internal validity? I also don't see how the author intend to focus (sort?) elements of guidelines into internal validity vs non-internal validity? Apologies if this is described in appendix C but I can't access it.
Author's response: This is indeed not an easy topic, and we have intensive discussions within our group on these questions. As a matter of fact, many items are not clearly and easily sorted to either internal or reproducibility or external validity, but may be considered gray areas. We throughout the manuscript deleted the misleading wording of investigating prevalence and impact of risk of bias, as we are rather interested in, e.g., primary literature in a side-note describing a risk of bias and a possible solution or control mechanism. This would fall under the category Ia (primary literature, not endorsed). A systematic review of experiments, while not focusing on guidelines, might include similar information (and would fall under the category IIIa). Appendix C lists formerly described sources of experimental guidelines (following Henderson et. al.) as our primary list to follow, as we assume most guidelines will include elements of that fall under the categories of this list.

Reporting
1. The ranking based on frequency of elements in the guidelines is technically part of the analysis and should be in the above section.
Author's response: We agree, the sentence was moved to the section above.

VERSION 2 - REVIEW

**REVIEWER 1**	Kristina Thayer<br>United States Environmental Protection Agency<br><br>Conflict of Interest: None declared
**REVIEW RETURNED**	21-05-18

**GENERAL COMMENTS**	Authors have been responsive to reviewer comments

**REVIEWER 2**	Marc Avey<br>ICF Canada<br><br>Conflict of Interest: None declared
**REVIEW RETURNED**	02-06-18

**GENERAL COMMENTS**	The protocol is much clearer with the wording changes which is extremely helpful given the complexity of the topic area. I look forward to reading the final publication.<br><br>Two minor quibbles for the authors to consider. 1) The list of societies to search in appendix B are exclusively professional neuroscientific organizations, but the protocol is more broadly focused on preclinical research in general. 2) The list of government organizaitons in appendix B appear to be exclusively academic funding agencies with no regulators (e.g. FDA).
